# Decanal Protects against UVB-Induced Photoaging in Human Dermal Fibroblasts via the cAMP Pathway

**DOI:** 10.3390/nu12051214

**Published:** 2020-04-25

**Authors:** Wesuk Kang, Dabin Choi, Taesun Park

**Affiliations:** Department of Food and Nutrition, Brain Korea 21 PLUS Project, Yonsei University, 50 Yonsei-ro, Seodaemun-gu, Seoul 120-749, Korea; wesuk42@naver.com (W.K.); vin1411@naver.com (D.C.)

**Keywords:** decanal, UVB, photoaging, cAMP, human dermal fibroblast

## Abstract

Solar ultraviolet (UV) radiation is the primary factor of cutaneous aging, resulting in coarse wrinkles and dryness. In this study, we aimed to test whether decanal, an aromatic compound found mainly in citrus fruits, inhibits UVB-mediated photoaging in human dermal fibroblasts and to explore whether its anti-photoaging effect occurs via cyclic adenosine monophosphate (cAMP) signaling. We found that decanal promotes collagen production dose-dependently. Meanwhile, it also increased the intracellular cAMP levels and decreased the number of molecules involved in the mitogen-activated protein kinase (MAPK)/activator protein 1 (AP-1) pathway, downregulating the collagen genes and upregulating the matrix metalloproteinase (MMP) genes in UVB-exposed dermal fibroblasts. Furthermore, it enhanced hyaluronic acid levels and hyaluronic acid synthase mRNA expression. Notably, the beneficial effects of decanal were lost in the presence of a cAMP inhibitor. Our results revealed the potential of decanal for preventing photoaging and suggested that its effects are cAMP-mediated in human dermal fibroblasts.

## 1. Introduction

Skin aging can be caused by intrinsic and extrinsic factors. Intrinsic aging is an unavoidable physiological step that leads to gradual dermal atrophy, whereas extrinsic aging is caused by environmental factors, including poor diet, stress and sun exposure, that lead to deep wrinkles, dry skin and loss of elasticity [1,2,3]. Solar ultraviolet (UV) radiation in particular, is a major factor accounting for most extrinsic aging; for this reason, extrinsic aging is also called photoaging [3]. UV irradiation causes damage to the dermal tissue. One of the hallmarks of this UV-induced damage of the dermal tissue is increased matrix metalloproteinase (MMP) expression, which causes collagen breakdown in the dermal fibroblast and promotes subsequent skin wrinkling [4,5].

Cyclic adenosine monophosphate (cAMP), one of the best-known secondary messengers, modulates various intracellular effects of neurotransmitters and hormones in numerous types of physiological processes [6,7,8]. Certain clues indicating that cAMP is an important regulator of collagen synthesis have been uncovered recently. For example, in human dermal fibroblasts, adenyl cyclase (Adcy) activator, forskolin and two other well-known cAMP-elevating agents (3-isobutyl-1-methylxanthine and dibutyryl cAMP) have also been found to inhibit MMP1 expression [9]. Similarly, a cAMP increase, triggered by the activation of a specific G-protein-coupled receptor (GPCR; an adenosine A2A receptor, upstream of Adcy), inhibits the MMP pathway, thus stimulating collagen production in human dermal fibroblasts [10]. Topical cilostazol (cAMP phosphodiesterase inhibitor) application protects against UVB-mediated MMP expression and subsequent photoaging, including wrinkle formation in the skin of hairless mice, through cAMP-dependent intracellular signaling [11].

Since procollagen reduction is a well-defined marker for photoaging in the dermal fibroblast [12,13,14], we tried to find the molecules that can enhance the procollagen level from our in-house phytochemical library. As a result of this screening, we identified that decanal caused a significant rise in the procollagen level. Decanal is an aromatic compound classified as an aldehyde with the chemical formula C_10_H_20_O and is found mainly in citrus fruits and other plants such as coriander (*Coriandrum sativum*), dill (*Anethum graveolens*) and catmint (*Nepeta cataria*) [15,16]. This compound has been shown to exhibit antifungal and antimicrobial properties [16,17]. However, the protective role decanal can play against skin aging has not yet been reported. Decanal has generally recognized as safe (GRAS) status and is approved for application in food as a flavoring agent by the Food and Drug Administration (FDA). The aim of this study was to investigate whether decanal attenuates UVB-induced photoaging and, if so, to clarify whether it exerts an anti-photoaging effect via cAMP-related signaling in human dermal fibroblasts.

## 2. Materials and Methods

### 2.1. Reagents

Human dermal fibroblast cell line (HS68) was obtained from American Type Culture Collection (Manassas, VA, USA). Dulbecco’s modified Eagle medium (DMEM) and fetal bovine serum (FBS) were obtained from HyClone (Logan, UT, USA), and penicillin-streptomycin was purchased from Gibco (Grand Island, NE, USA). Decanal, 3-(4,5-dimethylthiazol-2-yl)-2,5-diphenyltetrazolium bromide (MTT), dimethyl sulfoxide (DMSO) and SQ22536 were purchased from Sigma-Aldrich (St. Louis, MO, USA). The primary antibodies against protein kinase A catalytic subunit (PKA Cα), p38, p-p38, c-Jun N-terminal kinase (JNK), p-JNK, extracellular-signal-regulated kinase, p-ERK, c-Fos, p-c-Fos, c-Jun, p-c-Jun and glyceraldehyde 3-phosphate dehydrogenase (GAPDH) were sourced from Cell Signaling (Danvers, MA, USA). The horseradish-peroxidase-conjugated anti-rabbit IgG secondary antibody was purchased from Santa Cruz Biotechnology Inc. (Santa Cruz, CA, USA). Trizol and SuperScript reverse transcriptase were sourced from Invitrogen (Carlsbad, CA, USA). Bradford reagent, electrochemiluminescence (ECL) detection reagent and iQ SYBR green supermix were purchased from BioRad (Hercules, CA, USA). Bovine serum albumin (BSA) was purchased from MP Biomedicals (Irvine, CA, USA). Phosphate-buffered saline (PBS) was purchased from WelGENE (Daegu, Korea)

### 2.2. Cell Culture

Human foreskin fibroblast Hs68 cells were incubated in high-glucose Dulbecco’s modified Eagle medium supplemented with 10% (*v/v*) fetal bovine serum and 1% 100 U/mL penicillin-streptomycin at 37 °C in a 5% CO_2_ incubator (Sanyo, Osaka, Japan).

### 2.3. UV Irradiation

We used the UV cross-linker (Model CL-1000M; UVP, Upland, CA, USA) for UVB (280–320 nm) irradiation. The lamp was fixed 22 cm above the platform where the cultured cells were placed. The cells were then rinsed with PBS, covered with a thin layer of PBS and exposed to 20 mJ/cm^2^ UVB irradiation for 10 s.

### 2.4. Cell Viability Assay

Cell viability was evaluated by the MTT assay. Hs68 cells (8 × 10^3^ cells/well) were transferred into 96-well plates, pretreated with either the vehicle (DMSO) or 25–200 μM decanal and incubated for 24 h. The cultured cells were UVB-irradiated and cultured with either the vehicle or decanal for 24 h. Subsequently, plates were incubated with an MTT solution (3 mg/mL in PBS) for 3 h. The supernatant was then discarded, and purple formazan crystals were dissolved in DMSO. Absorbance was evaluated at 595 nm using Infinite M200 microplate reader (Tecan, Männedorf, Switzerland). In all the experiments, the final DMSO concentration was 0.1% (*v/v*), which has no effect on cell viability.

### 2.5. Procollagen Type I and Hyaluronic Acid Quantification

Hs68 cells (6 × 10^4^ cells/well) were seeded into 24-well plates, pretreated with either the vehicle or 25–100 μM decanal and cultured for 24 h. The cultured cells were then irradiated with UVB and treated with either the vehicle or decanal for 24 h. Procollagen type I and hyaluronic acid contents in the supernatants were then estimated using the procollagen I C-terminal peptide enzyme-linked immunosorbent assay (ELISA) kit (Takara, Shiga, Japan) and the Hyaluronan Quantikine ELISA kit (R&D Systems, Minneapolis, MN, USA), following the manufacturers’ protocols. Absorbance was measured at 590 nm using a microplate reader. The final procollagen type I and hyaluronic acid levels were normalized to the total cellular protein content. Where required, 50 μM SQ22536, a commercially available cAMP inhibitor, was added 1 h before decanal treatment.

### 2.6. cAMP Assay

Hs68 cells (6 × 10^4^ cells/well) were seeded into 24-well plates and incubated either with or without 50 μM decanal for 5, 15, 30, 45 and 60 min. The cells were then rinsed with PBS, lysed with HCl for 5 min and scraped off. The cAMP concentrations of the collected cell lysates were measured with the cAMP ELISA kit (Enzo, Plymouth Meeting, PA, USA) according to the manufacturer’s protocol. Absorbance was measured at 450 nm using a microplate reader. The cAMP content was normalized to the cellular protein level using Bradford assay, with bovine serum albumin used as a standard.

### 2.7. Western Blotting

Hs68 cells (7 × 10^5^ cells/dish) were seeded into 60-mm cell culture dishes, cultured in the presence or absence of 50 μM decanal, exposed to UVB as described previously and incubated for a further 30 min. The cells were lysed and harvested with a PRO-PREP protein extraction buffer (iNtRON, Seoul, Korea). The protein was separated by SDS-polyacrylamide gel electrophoresis prior to transferring to nitrocellulose membranes (Whatman, Dassel, Germany). The membranes were then blocked with BSA (5%) and incubated with 1:1000 diluted primary antibodies at 4 °C. The membranes were then incubated with the 1:5000 diluted corresponding secondary antibody for 1 h at 20 °C. The electrochemiluminescence detection reagent (ECL) was used to detect protein bands and the images were captured with Ez-capture (ATTO, Tokyo, Japan). The data were normalized to those for GAPDH.

### 2.8. Quantitative Reverse-Transcription PCR

Hs68 cells (2 × 10^5^ cells/well) were seeded into six-well plates, cultured for 6 h with either the vehicle or 50 μM decanal, irradiated with UVB and incubated with either the vehicle or decanal for an additional 6 h. When required, 50 μM SQ22536 was added 1 h before decanal treatment. Thereafter, the RNA in cells was isolated using Trizol and reverse transcribed using SuperScript IV reverse transcriptase to generate cDNA. PCR was then performed with iQ SYBR Green Supermix using CFX Real-Time System (BioRad). The relative gene levels were estimated by the threshold cycle (Ct) method, using GAPDH as the housekeeping gene. Details of the primer sets are given in Table 1.

### 2.9. Statistical Analysis

Results are shown as the mean ± standard error of mean (SEM). To determine any significant difference between the two groups, a Student’s *t*-test was conducted using the SPSS 25 software (SPSS; Chicago, IL, USA) with significance as *p* < 0.05.

## 3. Results

### 3.1. Decanal has Little Effect on Cell Viability in Hs68 Cells

**Figure 1:**
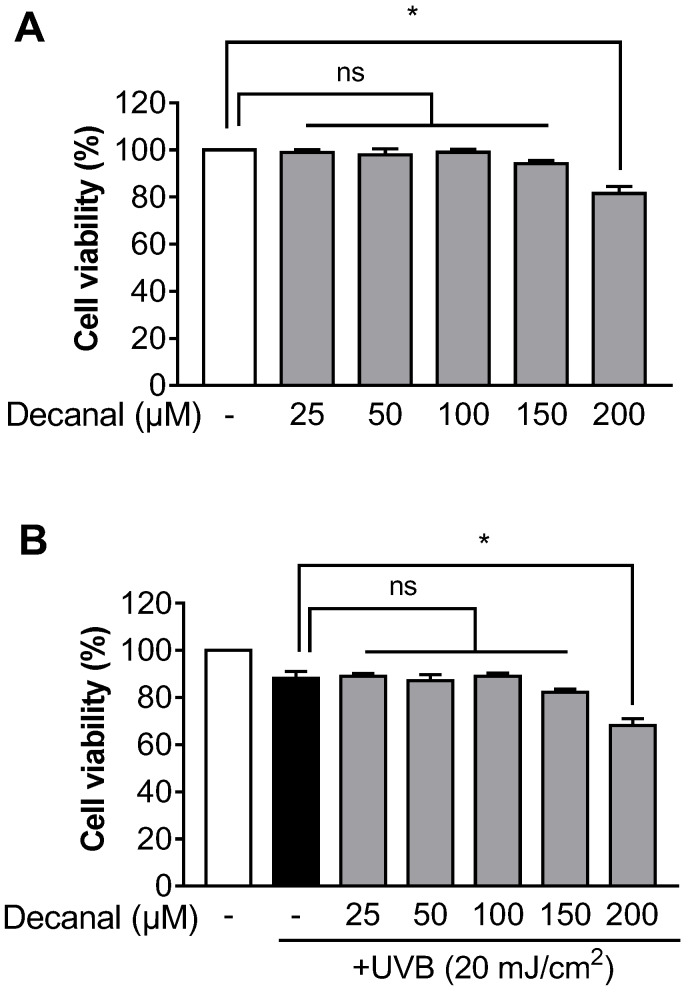
The effect of decanal on cell viability in Hs68 dermal fibroblasts. The cell viability of both (**A**) non-ultraviolet (UV) B-exposed and (**B**) UVB-exposed Hs68 cells was determined after incubation with either the vehicle or 25–200 μM decanal for 24 h. Data are shown as the mean ± SEM (*n* = 3). Significant differences are indicated as * *p* < 0.05.

To investigate the effect of decanal on cell viability, the MTT assay was performed. We found that up to 150 μM decanal showed no cytotoxicity to either non-UVB-exposed or UVB-exposed Hs68 cells. Cell viability was significantly decreased after treatment with 200 μM decanal (Figure 1A,B). Therefore, the maximum concentration of decanal used in the following experiments did not exceed 150 μM.

### 3.2. Decanal Attenuates UVB-Induced Collagen Degradation in Hs68 Cells

**Figure 2:**
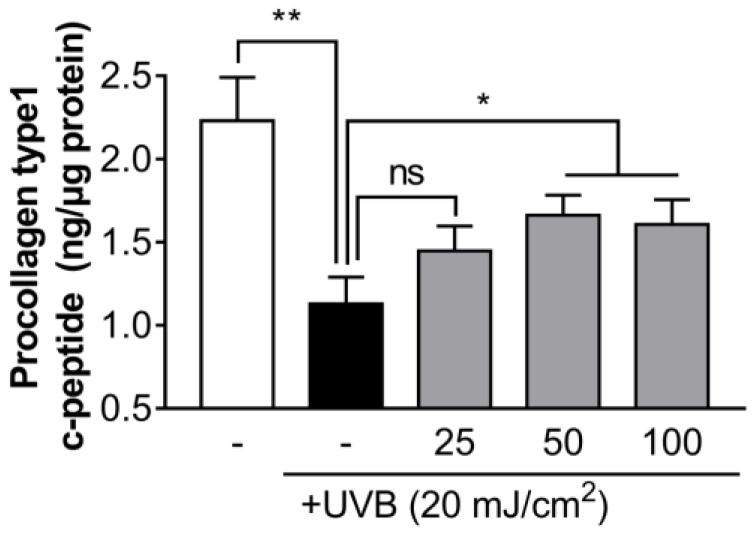
The effect of decanal on UVB-induced collagen degradation in Hs68 dermal fibroblasts. The collagen content was measured in the supernatant of Hs68 cells treated with either the vehicle or 25, 50 and 100 μM decanal for 24 h after UVB exposure. The final procollagen type I level was normalized to the total cellular protein content. Data are shown as the mean ± SEM (*n* = 3). Significant differences are indicated as * *p* < 0.05; ** *p* < 0.01.

We evaluated whether decanal can inhibit collagen degradation in UVB-irradiated Hs68 cells. Decanal treatment significantly inhibited the UVB-induced procollagen reduction dose-dependently, and this effect appeared to saturate at around 50 µM decanal treatment (Figure 2). Based on these results, all further experiments were carried out with 50 μM decanal concentration.

### 3.3. Decanal Upregulated cAMP/PKA Signaling Pathway in Hs68 Cells

**Figure 3:**
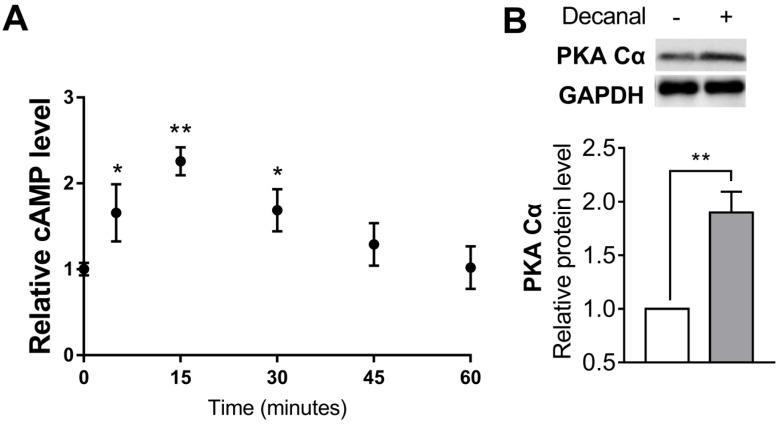
The effect of decanal on the cyclic adenosine monophosphate–protein kinase A (cAMP-PKA) pathway in Hs68 dermal fibroblasts. (**A**) The time course of decanal-induced intracellular cAMP levels was determined after incubation with either the vehicle or 50 μM decanal for 5, 15, 30, 45 and 60 min. (**B**) The protein expression of protein kinase A catalytic subunit (PKA Cα) was measured after incubation with 50 μM decanal for 30 min. Data are shown as the mean ± SEM (*n* = 3). Significant differences between decanal and non-stimulated control groups are shown as * *p* < 0.05; ** *p* < 0.01.

In order to examine the involvement of the cAMP/PKA pathway in Hs68 cells, we assessed the intracellular cAMP levels after 50 µM decanal treatment time-dependently. The peak increase of cAMP levels was seen at 15 min, and the concentration returned to the basal value within 60 min (Figure 3A). Furthermore, decanal significantly increased the protein level of PKA Cα, which is mainly activated by cAMP (Figure 3B).

### 3.4. Decanal Inhibits UVB-Induced MAPK Pathway in Hs68 Cells

**Figure 4:**
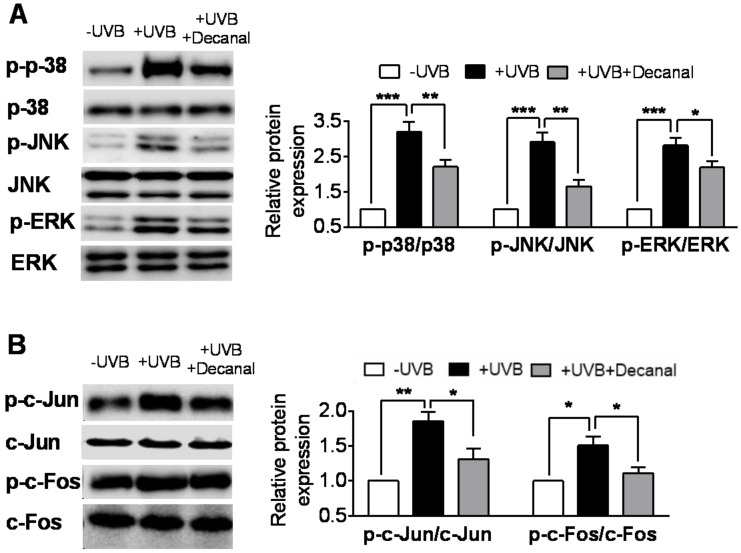
The effect of decanal on the UVB-induced mitogen-activated protein kinase (MAPK) pathway in Hs68 dermal fibroblasts. Hs68 cells were treated with either decanal or the vehicle and exposed to UVB. (**A**) Phosphorylation of MAPK proteins (p38, c-Jun N-terminal kinase (JNK) and extracellular-signal-regulated kinase (ERK)) and (**B**) c-Jun and c-Fos (MAPK downstream molecules) were determined. Data are shown as the mean ± SEM (*n* = 3). Significant differences are indicated as * *p* < 0.05; ** *p* < 0.01; *** *p* < 0.001.

We then examined the involvement of MAPK pathway in Hs68 cells. In our experiments, UVB irradiation significantly promoted the phosphorylation of MAPK proteins (p38, JNK and ERK). Compared to the UVB-irradiated group, the addition of 50 µM decanal significantly suppressed UVB-induced MAPK protein phosphorylation (Figure 4A). We also investigated the phosphorylation of activator protein 1 (AP-1) proteins (c-Fos and c-Jun), which are MAPK downstream molecules. UVB irradiation led to AP-1 protein phosphorylation. Decanal significantly inhibited UVB-induced phosphorylation of AP-1 proteins (Figure 4B).

### 3.5. Decanal Promotes Collagen Synthesis Via cAMP Signaling Pathway

**Figure 5:**
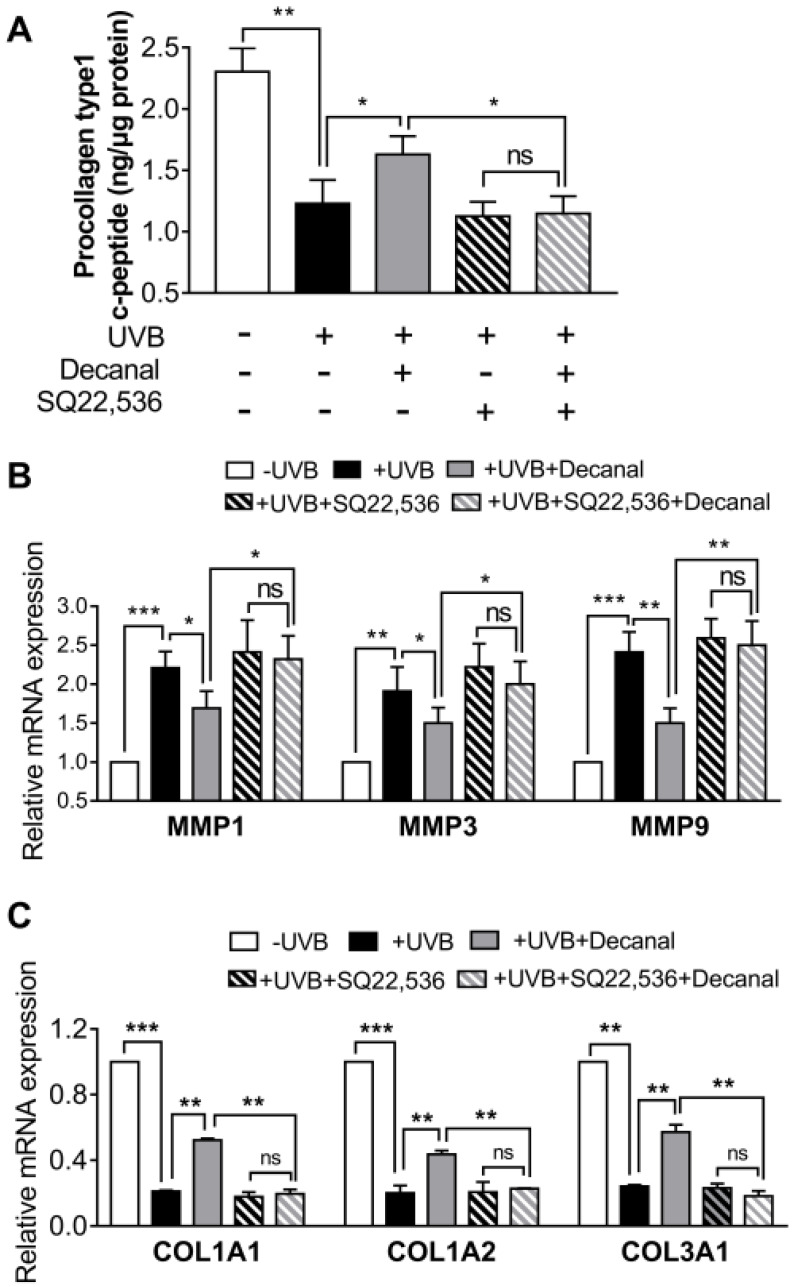
The effect of decanal on collagen synthesis via the cAMP pathway. Hs68 cells were treated with decanal or the vehicle and exposed to UVB. The SQ22536 or the vehicle was pretreated for 1 h before the decanal treatment. (**A**) Collagen contents; (**B**) mRNA expression of matrix metalloproteinase (*MMP)* 1, *MMP3* and *MMP9*; and (**C**) mRNA expression of collagen type I alpha 1 chain (*COL1A1*), *COL1A2* and *COL3A1* were analyzed. The final procollagen type I level was normalized to the total cellular protein content. Data are shown as the mean ± SEM (*n* = 3). Significant differences are indicated as * *p* < 0.05; ** *p* < 0.01; *** *p* < 0.001.

In order to further investigate whether decanal-induced collagen synthesis was mediated via the cAMP signaling pathway, some cells were pretreated with 50 µM SQ22536 for 1 h before decanal treatment. The SQ22536 pretreatment completely blocked decanal-induced collagen production in UVB-exposed Hs68 cells (Figure 5A). Then we assessed the effect of decanal on gene expression in UVB-exposed Hs68 cells. Decanal significantly reduced UVB-induced mRNA expression of *MMP1*, *3* and *9* (Figure 5B). In contrast, the decanal treatment significantly increased mRNA expression of collagen type I alpha 1 chain *(COL1A1)*, *COL1A2* and *COL3A1* as compared to that in UVB-irradiated cells (Figure 5C). Under these conditions, the SQ22536 treatment significantly blocked the inhibitory effects of decanal on the *MMP1, 3* and *9* mRNA levels (Figure 5B) and the stimulatory effects of decanal on *COL1A1*, *COL1A2* and *COL3A1* mRNA levels (Figure 5C).

### 3.6. Decanal Enhances Hyaluronic Acid Synthesis Via cAMP Signaling Pathway

**Figure 6:**
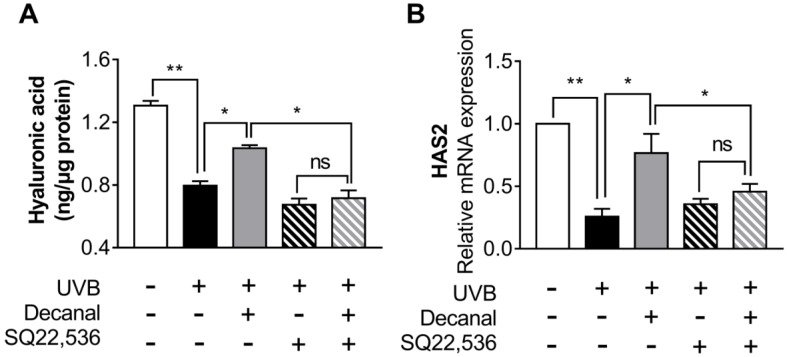
The effect of decanal on hyaluronic acid synthesis via the cAMP signaling pathway. Hs68 cells were treated with either decanal or the vehicle and exposed to UVB. Other samples were pretreated with either SQ22536 or the vehicle for 1 h before decanal treatment. (**A**) Hyaluronic acid concentrations and (**B**) the mRNA expression of *HAS2* were examined. The final hyaluronic acid levels were normalized to the total cellular protein content. Data are shown as the mean ±SEM (*n* = 3). Significant differences are indicated as * *p* < 0.05; ** *p* < 0.01.

**Figure 7:**
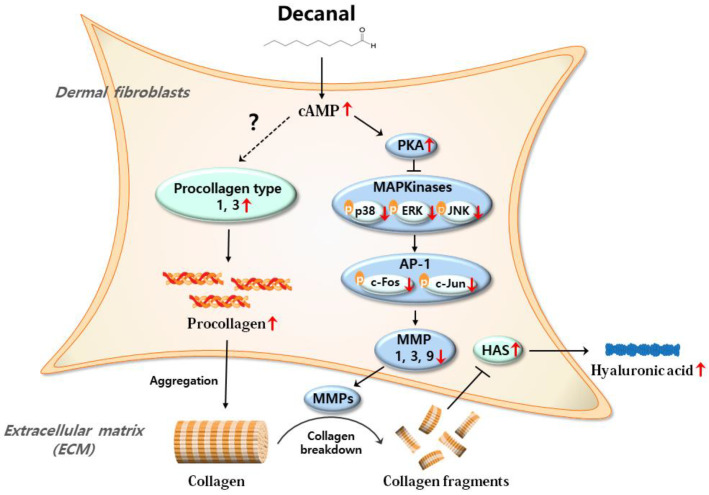
The proposed mechanism by which decanal protects photoaging in dermal fibroblasts. As depicted, higher production of cAMP in response to decanal increases the procollagen levels. The mechanism of cAMP-mediated procollagen production is not yet fully defined. On the other hand, cAMP production in response to decanal decreases the MMPs levels, with the possible involvement of the PKA/MAPK/AP-1 pathway. Then, collagen is less cleaved by decreased MMPs, resulting in increased hyaluronan synthase 2 (HAS2) gene expression and hyaluronic acid levels.

We also assessed whether decanal promotes hyaluronic acid synthesis via the cAMP pathway. Decanal significantly increased hyaluronic acid synthesis, but a 50 µM SQ22536 treatment significantly suppressed decanal-induced hyaluronic acid synthesis in UVB-irradiated Hs68 cells (Figure 6A). We then estimated the effect of decanal on gene expression in UVB-irradiated Hs68 cells. Decanal significantly increased the gene expression of hyaluronic acid synthase 2 (*HAS2*), whereas these effects of decanal were lost in the presence of SQ22536 (Figure 6B). Taken together, decanal-induced activation of the cAMP pathway is the likely mechanism underlying the anti-photoaging effects of decanal in human dermal fibroblasts (Figure 7).

## 4. Discussion

In this study, UVB was used to induce experimental photoaging in human dermal fibroblasts. The UV region is classified by three zones: UVA (320–400 nm), UVB (280–320 nm) and UVC (180–280 nm). The ozone layer blocks UVC. Although there is around 20 times more UVA than UVB in terrestrial solar light, it is well-known that UVB is the primary factor underlying UV-induced photoaging due to its higher energy [18]. Indeed, UVB and UVA can both interact with endogenous photosensitizers and chromophores, resulting in reactive oxygen species production that causes damage to DNA and proteins; however, only UVB can directly affect DNA, synthesizing photoproducts including pyrimidine–pyrimidine dimers [19,20]. UVB, which is more harmful than UVA, effectively produces sunburn and transient inflammatory reactions, thus causing the skin remodeling that results in the visible photoaging symptoms [21,22,23]. Although this study was performed in dermal fibroblasts, given that epidermal keratinocytes would be more vulnerable to UV exposure than dermal fibroblasts, it would be intriguing to explore the beneficial effects of decanal in epidermal keratinocytes in the future.

Collagen fragmentation is a crucial step that induces photoaging and can be reached by excessive MMP expression in response to UVB exposure [24]. UVB is known to promote the expression of several MMPs, such as MMP1, 3 and 9, in the human skin [5,25]. It has been recently reviewed that almost all extracellular matrix components can be degraded by the combined action of MMPs, with distinct specificities for each MMP. Within the collagen triple helix, MMP1 degrades fibrillar collagens, which are mainly type I and III collagens (that together account for 90% of the total collagen), at a single site. After being degraded by MMP1, fibrillar collagen is further cleaved by MMP3 and MMP9 [26,27,28,29]. In this study, the mRNA expression of the three key MMPs (MMP1, 3 and 9) was shown to be inhibited by decanal in UVB-exposed dermal fibroblasts.

The cAMP systems have previously been found to work as an all-or-none switch in fibroblasts/fibroblast-like cells in various tissues [30,31,32]. For example, lower levels of cAMP boost cardiac fibroblast migration, while higher cAMP levels inhibit such migration [30]. Similarly, it is possible that cAMP acts as a switch for collagen production in the dermal fibroblast where different concentrations exert opposite effects. Perez-Aso et al. found that physiological levels of cAMP (150% of control), resulting from pharmacologic adenosine A2A receptor stimulation, promote collagen production, whereas high levels of cAMP (15,689% of control), beyond the physiological range that can be achieved by direct and irreversible activation of Adcy, inhibit collagen production [10]. In this study, by using a specific cAMP inhibitor, SQ22536, we clearly demonstrated that a modest cAMP increase (225% of control), elicited by decanal treatment, inhibited collagen degradation in UVB-irradiated dermal fibroblasts.

We demonstrated that decanal treatment in UVB-irradiated dermal fibroblasts attenuated the decrease in hyaluronic acid levels. Hyaluronic acid is the key molecule involved in skin hydration [33,34]. It is synthesized on the membrane by hyaluronic acid synthases and, among them, HAS2 seems to be the main subtype in the dermal fibroblasts, based on the reports that the extent of HAS2 degradation is tightly associated with hyaluronic acid decrease [35,36,37]. Although the causes for HAS2 downregulation during photoaging are largely unknown, collagen degradation is regarded as a possible factor for hyaluronic acid decrease in reaction to UVB. It was reported that collagen degradation generates bioactive collagen fragments, which activate αvβ3-integrin signaling and subsequently lead to HAS2 downregulation [36].

In this study, decanal treatment activated the cAMP signaling pathway, leading to a subsequent decrease in MAPK/AP-1 activation and downstream genes (e.g., *MMP1*, *3* and *9*). Although mechanisms of how decanal increases intracellular cAMP levels remain unknown, it is noteworthy that decanal is known as a ligand of various olfactory receptors (ORs), including OR1G1, OR52D1 and OR2W1, which belong to a large GPCR family [38,39]. GPCR activation in the membrane further stimulates Adcy that converts ATP to cAMP [40,41]. Further investigation is needed to reveal the exact mechanism of decanal responsible for triggering the cAMP pathway.

It is well-known that intracellular reactive oxygen species (ROS) generation after UV irradiation is also responsible for photoaging [42,43]. Given that decanal exhibits antioxidant activity in HeLa cell lines [16], we cannot exclude the possibility that the antioxidant property of decanal could also contribute to preventing UVB-induced photoaging in dermal fibroblasts.

## 5. Conclusions

In summary, we demonstrated that decanal markedly attenuates photoaging through cAMP-mediated signaling in human dermal fibroblasts. Our results revealed the potential of decanal for the prevention of photoaging and proposed that its effects are cAMP-mediated in human dermal fibroblasts. Further research is required to determine the potential for developing decanal as an anti-skin-aging agent in cosmetics, skin products and functional foods.

## Figures and Tables

**Table 1:** The primer sequences.

Type	Gene Description	Sequences (5′→3)
Human	Collagen type I alpha 1 chain (*COL1A1*)	F: ACATGTTCAGCTTTGTGGACC
		R: TGTACGCAGGTGATTGGTGG
	Collagen type I alpha 2 chain (*COL1A2*)	F: CGGACTTTGTTGCTGCTTGC
		R: CAGCAAAGTTCCCACCGAGA
	Collagen type III alpha 1 chain (*COL3A1*)	F: TCGAGGCAGTGATGGTCAAC
		R: AGGTCCAACTTCACCCTTAGCA
	Matrix metalloproteinase 1 (*MMP1*)	F: AGGGGAGATCATCGGGACAACTC
		R: GAGAGTCCAAGAGAATGGCCGA
	Matrix metalloproteinase 3 (*MMP3*)	F: GCATTCAGTCCCTCTATGGACCTC
		R: GGGATTTGCGCCAAAAGTGC
	Matrix metalloproteinase 9 (*MMP9*)	F: GACGCAGACATCGTCATCCA
		R: AAACCGAGTTGGAACCACGAC
	Hyaluronic acid synthase 2 (*HAS2*)	F: GAGCAGCCCATTGAACCAGA
		R: AGGAAGCGCAGAATTGGGAG
	Glyceraldehyde-3-phosphate dehydrogenase (*GAPDH*)	F: GACAGTCAGCCGCATCTTCT
		R: GCGCCCAATACGACCAAATC
